# Marginal Adaptation of BioRoot RCS, MTA-Fillapex, EpoxySeal, and Sealapex to Radicular Dentin: An In Vitro Study

**DOI:** 10.7759/cureus.64380

**Published:** 2024-07-12

**Authors:** M Kirthiga, George Thomas, Sunil Jose, Sona Joseph, Manju Krishna

**Affiliations:** 1 Department of Conservative Dentistry and Endodontics, Mahe Institute of Dental Sciences and Hospital, Puducherry, IND

**Keywords:** sealapex, mta fillapex, marginal adaptation, epoxy seal, bio root rcs

## Abstract

Introduction

The primary goal of endodontic therapy is to achieve a three-dimensional filling of the root canal. The sealer plays a crucial role in filling the residual gaps between the gutta-percha and the canal wall, which prevents fluids and bacteria from entering the canal and causing complications. This study evaluates and compares the sealing ability of four root canal sealers to the root dentin.

Methods and materials

In this study, forty single-rooted mandibular premolar teeth with fully-formed apices were collected. The teeth were decoronated and instrumented. Later, the teeth were randomly divided into four groups, each comprising ten specimens, based on the sealer used for obturation. The first group was obturated with BioRoot RCS (Septodont, Septodont Holding, Paris, France), the second group with MTA-Fillapex (Angelus, Angelus Indústria de Produtos Odontológicos S/A, Brazil), the third group with EpoxySeal (Safe Endo, SafeEndo Dental India Pvt. Ltd., Gujarat, India), and the fourth group with Sealapex (Kerr, Kerr Corporation, Brea, CA). Following obturation, the teeth were sectioned vertically using a diamond disc, and the marginal adaptation of these sealers to the root dentin was assessed using scanning electron microscopy (SEM). The values were then statistically analyzed.

Results

EpoxySeal showed the maximum amount of marginal adaptation (5.22±0.47), followed by BioRoot RCS (5.48±0.58) and MTA-Fillapex (8.24±0.74), and the least amount of marginal adaptation was shown by Sealapex (11.64±1.35). Based on the analysis of variance (ANOVA), a statistically significant difference (p≤0.001) was observed. According to Tukey's post hoc test, the mean difference between all groups showed statistical significance (p≤0.05) except between BioRoot RCS and EpoxySeal.

Conclusion

Within the limitations of this in vitro study, it is concluded that EpoxySeal and BioRoot RCS exhibited superior marginal adaptation to the root dentin. BioRoot RCS can be recommended as a sealer of choice owing to its additional properties, such as antimicrobial and hydrophilic affinity during setting.

## Introduction

The focus of endodontic treatment is to disinfect the root canal space and seal it with a three-dimensional filling material, thereby preventing reinfection. Often, this objective is not fulfilled due to the complex anatomical variations in the root canal and the resistance of microorganisms to conventional chemo-mechanical disinfection methods. The residual microorganisms in the untreated root canal space can lead to secondary infections and further inflammation of the periapical space [1].

An optimal root canal sealer should efficiently penetrate the untreated spaces within the root canal and establish a hermetic seal, effectively addressing any voids in conjunction with the primary core material [2]. Since gutta-percha offers little or no bond to root canal walls, the sealer can bond to root canal dentin, which becomes the most critical aspect in multidimensional obturation [3]. Hence, sealers play a crucial role in facilitating the binding, lubrication, and sealing of gutta-percha, as well as in the plugging of the lateral canal [4]. So an ideal sealer must form a strong interface with the substrate. This interface should remain dimensionally stable with minimal shrinkage or expansion [5].

Some of the traditional sealers used for obturation are based on zinc oxide, eugenol, and calcium hydroxide. These materials have less-than-ideal properties as sealers, and often their disadvantages outweigh the advantages. Then epoxy resin-based sealers were introduced. Epoxy resin is recognized as the gold standard [6]. The mechanical bond between the resin sealer and the root dentin is increased because it binds to the dentin and penetrates the microirregularities more effectively [7]. Later, mineral trioxide aggregate (MTA)-based sealers were introduced with many favorable properties, including bio-inertness and antimicrobial properties. These materials offered good sealing ability and adhesion to root dentin. Further developments in the field of sealers led to the introduction of bioactive sealers. The more recent calcium silicate-based sealers offer numerous advantages over traditional sealers such as calcium hydroxide and zinc oxide eugenol.

This study aims to evaluate the sealing ability of four currently available root dentin sealers. The sealers tested are BioRoot RCS, MTA-Fillapex, EpoxySeal, and Sealapex. BioRoot RCS is based on calcium silicate, MTA-Fillapex is based on MTA, EpoxySeal is based on resin, and Sealapex is based on calcium hydroxide.

## Materials and methods

The study was conducted at the Mahe Institute of Dental Sciences and Hospitals, Chalakkara, Mahe, Puducherry, India. Forty straight, single-rooted, intact mandibular premolar teeth were collected for the study, which were extracted for orthodontic reasons.

Inclusion criteria

Mandibular premolars with intact root apices with single root canals were included.

Exclusion criteria

Teeth with root fractures, caries, restoration, and resorption were excluded in this study.

Grouping

We randomly divided the 40 samples into four groups (ten samples each): Group I (BioRoot RCS (Septodent, Septodont Holding, Paris, France)); Group II (MTA-Fillapex (Angelus, Angelus Indústria de Produtos Odontológicos S/A, Brazil)); Group III (EpoxySeal (Safe Endo, SafeEndo Dental India Pvt. Ltd., Gujarat, India)); and Group IV (Sealapex (Kerr, Kerr Corporation, Brea, CA)).

Specimen preparation

The specimens were decoronated using a diamond disc to standardize the root canal length at 14 mm. Access opening was done using endoacess bur, and working length determination was done using the No. 10 K file. The root canals were enlarged using Protaper (Niti) files till F3, and the canals were irrigated with 5.25% sodium hypochlorite (NaOCl) and saline. The tooth samples were subsequently divided into four groups (n=10) based on the type of sealer used to fill the root canals.

Obturation of the root canals

**Figure 1:**
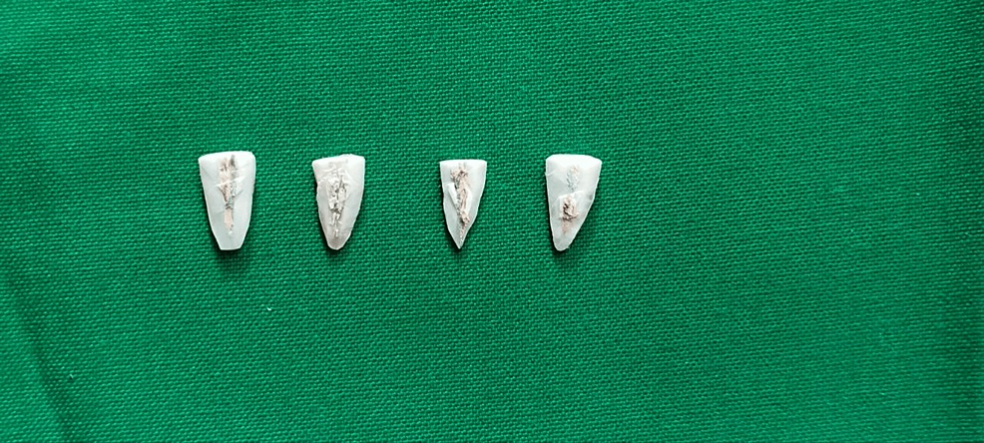
Vertically sectioned representative samples after obturation

The canals were irrigated with 17% ethylenediaminetetraacetic acid (EDTA) as a final rinse and dried using paper points before starting with obturation. The sealers were prepared following the manufacturer's guidelines. No. 35 GP mastercone was selected, coated with a sealer, and placed inside the root canal, followed by lateral compaction with accessory gutta-percha points using a finger spreader. In all samples, obturating material was removed 3 mm below the cementum-enamel junction and replaced with Cavit G. The specimens were then placed in a humidifier, where they were exposed to over 95% relative humidity at 37°C for 10 days. After this period, all samples underwent vertical sectioning using a diamond disc in a slow-speed handpiece (Figure 1).

Preparation for scanning electron microscopic analysis

**Figure 2:**
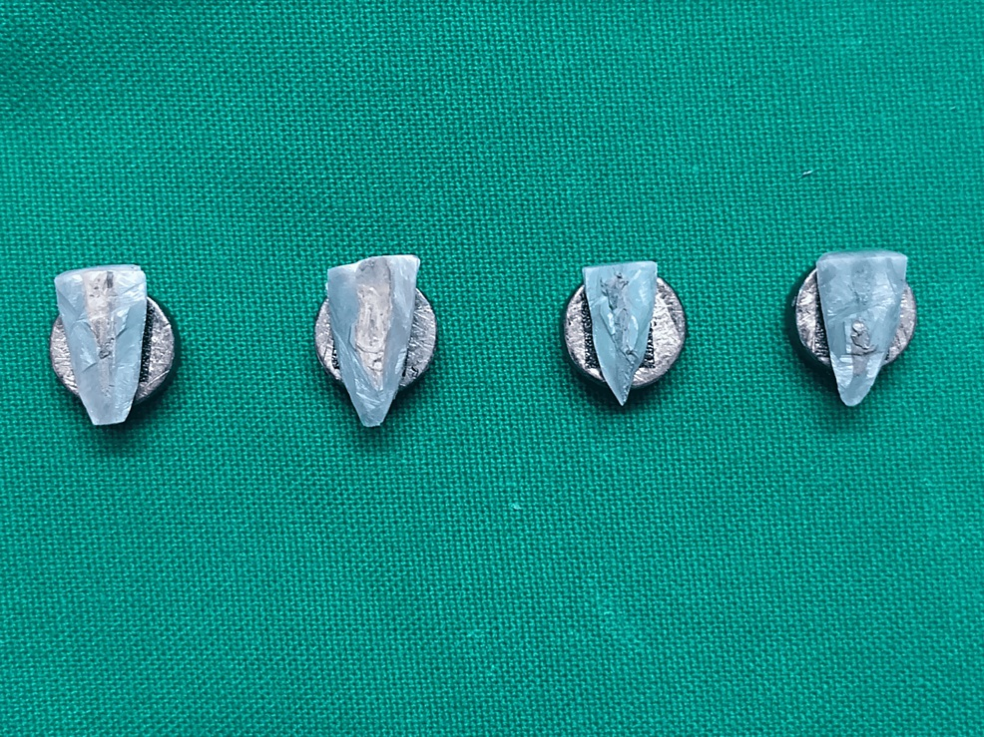
Gold sputter coated representative samples mounted on to aluminium stub

**Figure 3:**
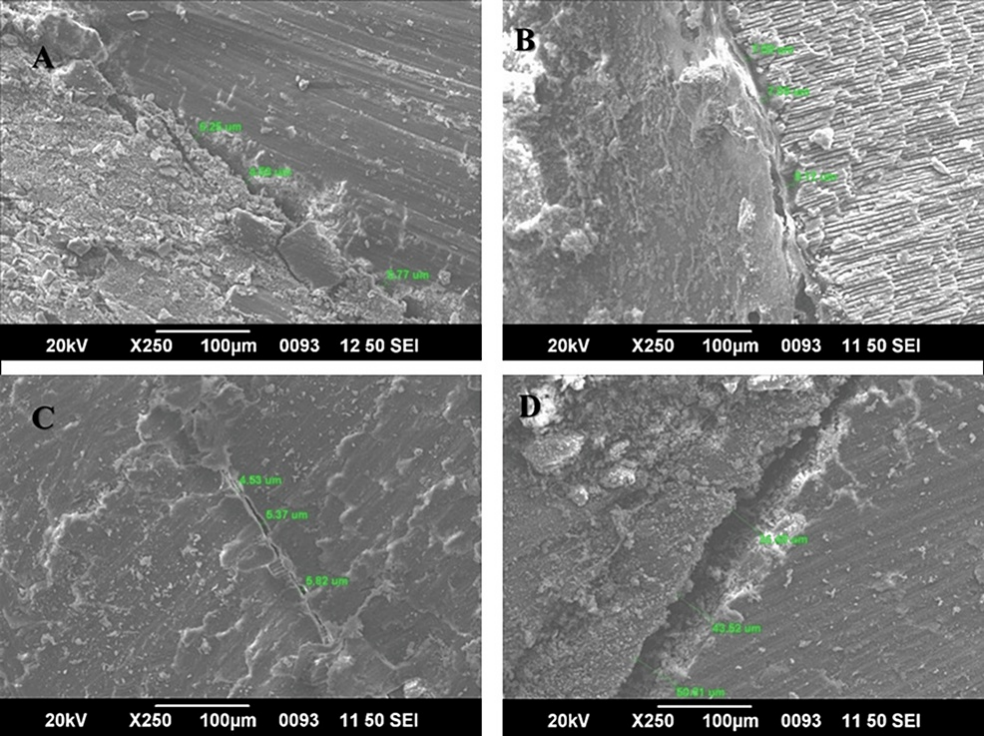
Scanning electron microscopy images A: BioRoot RCS; B: MTA-Fillapex; C: EpoxySeal; D: Sealapex

The specimens from each group were affixed to an aluminum stub, with the entire root canal prominently visible and oriented upwards. Each specimen was then coated with a thin layer of gold in a gold sputter coating machine (Figure 2). Subsequently, the samples were examined using a scanning electron microscope at ×200 magnification, and photographs of representative sections were captured (Figure 3).

Statistical analysis

The results were statistically analyzed using IBM SPSS Statistics for Windows, Version 26 (Released 2019; IBM Corp., Armonk, New York, United States). A one-way analysis of variance (ANOVA) test and Tukey's post hoc analysis were used to compare the gap formation (in μm) between four sealers.

## Results

Each experimental group underwent an evaluation to assess gap formation using four different types of sealers. Sealer penetration was determined by measuring the distance from the sealer-gutta-percha interface to the dentinal wall in a micrometer for each sample group (n=10) at a magnification of 200X using scanning electron microscope images. The results presented in Table 1 illustrate the mean and standard deviation of the gap formation at the gutta-percha-sealer interface to the dentinal wall for each group (BioRoot RCS, MTA-Fillapex, EpoxySeal, and Sealapex). Sealapex exhibited the highest mean value of 5.48±0.58, followed by MTA-Fillapex at 8.24±0.74, BioRoot RCS at 5.48±0.58, and EpoxySeal at 5.22±0.47. These findings indicate that the epoxy resin-based sealer demonstrated the highest level of marginal adaptation, followed by BioRoot RCS and MTA-Fillapex, while Sealapex showed the least marginal adaptation at 11.64±1.35. The mean values obtained by one-way ANOVA indicated a high level of significance for the gap formation between the sealer and root dentinal wall interface (p<0.01) (Table 1).

**Table 1:** Descriptive statistics and one way-ANOVA test *significance inferred at p≤0.05 based on one way-ANOVA SD: standard deviation; ANOVA: analysis of variance

Group	N	Mean ± SD	95% Confidence Interval for Mean		Minimum	Maximum	F value	p-value*
			Lower Bound	Upper Bound				
BioRoot RCS	10	5.48 ± 0.58	5.07	5.90	4.53	6.25	121.89	0.001
MTA-Fillapex	10	8.24 ±0.74	7.71	8.77	7.45	9.12		
EpoxySeal	10	5.22 ±0.47	4.89	5.56	4.53	5.89		
Sealapex	10	11.64±1.35	10.67	12.60	9.89	14.21		

According to post-hoc multiple comparisons of groups based on gap formation between the sealers and root dentin, we found statistically significant differences between BioRoot RCS and Sealapex (p-value=0.001), BioRoot RCS and MTA-Fillapex (p-value=0.001), groups MTA-Fillapex and Sealapex (p-value=0.001), group MTA-Fillapex and EpoxySeal (p-value=0.001), EpoxySeal and Sealapex (p-value=0.001) except between BioRoot RCS and EpoxySeal (p-value=0.90) which is not statistically significant (Table 2). The Tukey's test reveals that Group III (EpoxySeal) produces the highest marginal adaptation to the dentinal wall and differs significantly from other groups except Group II (BioRoot RCS), while Group IV (Sealapex) has the lowest marginal adaptation to root dentin and differs significantly from other groups as well (Table 2).

**Table 2:** Post hoc test using Tukey honestly significant difference (HSD) *. The mean difference is significant at the 0.05 level.

Multiple Comparisons- Tukey HSD						
(I) Group	(J) Group	Mean Difference (I-J)	Std. Error	p-value*	95% Confidence Interval	
					Lower Bound	Upper Bound
BioRoot RCS	MTA-Fillapex	-2.75^*^	0.38	0.001	-3.79	-1.72
	EpoxySeal	0.26	0.38	0.90	-.77	1.29
	Sealapex	-6.15^*^	0.38	0.001	-7.18	-5.12
MTA-Fillapex	BioRoot RCS	2.75^*^	0.38	0.001	1.72	3.79
	EpoxySeal	3.02^*^	0.38	0.001	1.99	4.05
	Sealapex	-3.40^*^	0.38	0.001	-4.43	-2.37
EpoxySeal	BioRoot RCS	-0.26	0.38	0.90	-1.29	0.77
	MTA-Fillapex	-3.02^*^	0.38	0.001	-4.05	-1.99
	Sealapex	-6.42^*^	0.38	0.001	-7.45	-5.38
Sealapex	BioRoot RCS	6.15^*^	0.38	0.001	5.12	7.18
	MTA-Fillapex	3.40^*^	0.38	0.001	2.37	4.43
	EpoxySeal	6.42^*^	0.38	0.001	5.38	7.45

**Figure 4:**
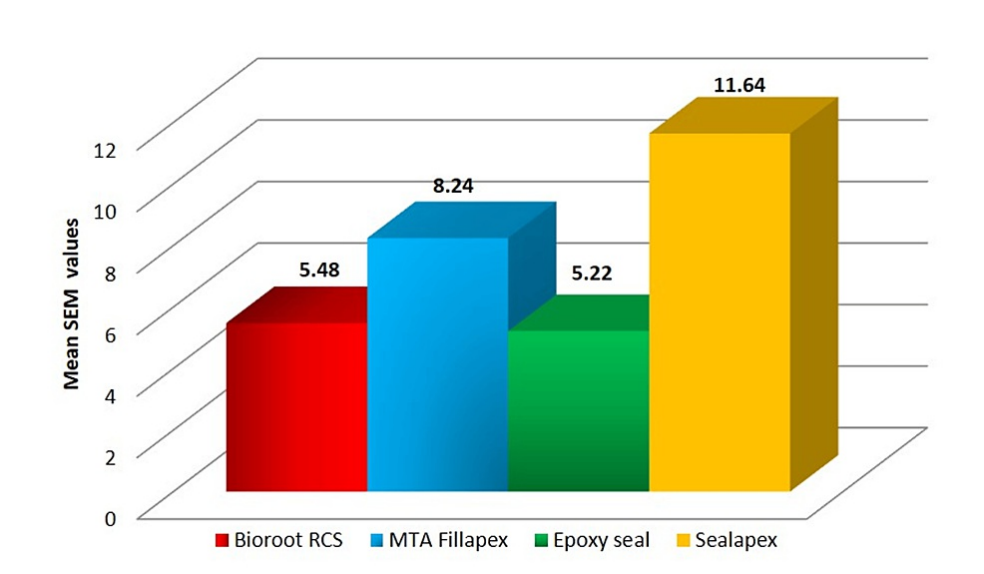
Graphical representation of gap formation SEM: scanning electron microscopy

The graph illustrates that EpoxySeal (5.22) exhibited the least amount of gap formation to radicular dentin, followed by BioRoot RCS (5.48), MTA-Fillapex (8.24), and Sealapex (11.64) (Figure 4).

## Discussion

The effective instrumentation of root canals is an essential aspect of successful endodontic treatment. This allows the canal space to be shaped and ready for filling with an inert substance, which reduces or eliminates the possibility of reinfection [8]. The primary objective of this three-dimensional obturation is to achieve a fluid-tight seal throughout the entire root canal system, which is essential for preventing both oral and apical microleakage [9]. Endodontic failure is frequently caused by a prolonged microbiological infection [10]. It is challenging to completely eradicate the germs in the root canals due to variations in the anatomical characteristics of the root canal system, such as fins, isthmi, and auxiliary canals. Various intracanal irrigants and medicaments, including sodium hypochlorite, calcium hydroxide, sodium hydroxide, and chlorhexidine, are utilized in the treatment of the root canal in addition to mechanical cleaning to remove germs in the debridement of the root canal. However, residual germs could still be present in the root canal system. Consequently, to confine and eventually eradicate any remaining bacteria within the filled root canal, a hermetic closure of the root canal space is necessary using sealers [11].

Commercial sealers are classified into zinc oxide eugenol, calcium hydroxide, glass ionomer, resin, silicone, and calcium silicate-based sealers [12]. These sealers are used with the traditional lateral condensation technique for obturating the root canal. In the present study, sealers were also used with the lateral condensation technique [13]. Gutta-percha is a preferred material for effective obturation when combined with a root canal sealant. Nevertheless, the application of gutta-percha points alone is inadequate for sealing all gaps, including those found between dentinal walls, abnormalities in root canals, and lateral and accessory canals. Hence, a sealer is required to seal the voids between the gutta-percha and root canal, which has been shown to be the preferred material for successful obturation. The utilization of a sealer is imperative to achieve optimal results. It ensures that all gaps are filled and the root canal is effectively sealed [14].

This in vitro study investigated the sealing ability of root canal sealers Sealapex (Kerr), MTA-Fillapex (Angelus), BioRoot RCS (Septodont), and Epoxy Seal (Safe Endo) to dentin using a scanning electron microscope (SEM). The significant advantage of SEM over other methods is its capability to observe submicron-level defects at the desired magnification and preserve microphotographs for subsequent evaluation, thus contributing to a comprehensive and accurate analysis. SEM provides a more accurate and precise evaluation of marginal adaptation, making it a superior method for evaluating sealing ability [15].

Based on the results of this study, epoxy resin-based sealers showed lesser gap formation at the dentin sealer interface, whereas selapex sealers showed the maximum gap formation. Epoxy resin-based sealers in root canal therapy have been found to offer several benefits over other types of sealers. Specifically, these sealers have exhibited reduced solubility, improved apical seal, and enhanced microretention for root canal dentin [16]. The catalyst in epoxy resin sealers is an epoxy compound, which also provides excellent texture tolerance, long-term dimensional stability, and biocompatibility [17]. Dadpe AS et al. stated that epoxy resin-based sealers exhibit good flow and adequate adhesive properties to root dentin, which can be achieved through the formation of covalent bonds with collagen, facilitated by the chemical bonding of epoxy resin [18].

BioRoot RCS is a root canal sealer based on tricalcium silicate. The interaction between dentine fluid and BioRoot RCS has the potential to promote biomineralization. This process can result in the formation of mineral plugs inside dentinal tubules, thereby enhancing the biological activity within the root canal [19]. In this study, there were some marginal gaps observed between the BioRoot RCS and root dentin. According to studies, it has been reported that the application of EDTA has been found to decrease the wetting of dentine surfaces since bio-ceramic-based sealers possess hydrophilic properties, which is in contrast to epoxy-based sealers that are hydrophobic. This makes epoxy resin sealer more suitable for an acidic medium, such as EDTA [20,21].

MTA-Fillapex is a salicylate resin- and calcium silicate-based sealer. MTA-Fillapex showed a minimum marginal adaptation compared to that of the epoxy seal and BioRoot RCS. In a study conducted by Reyes-Carmona et al., it was reported that the apatite produced by MTA and phosphate-buffered saline was deposited within collagen fibrils, which facilitated mineral nucleation on dentine and led to the formation of an interfacial layer with tag-like structures [22]. The result of our study is similar to a study conducted by Sagsen et al., where they found that MTA-Fillapex demonstrated the lowest push-out bond strength (PBS) to root dentin as compared to epoxy resin and calcium silicate-based sealers, which might be due to the improper adaptation of those resin tags to root dentin [23].

Sealapex demonstrated the least amount of marginal adaptation due to its extended setting time and high solubility, potentially resulting in a non-uniform setting reaction and the formation of a poorly developed matrix. It also shows significant sorption and volumetric expansion in a 100% humid environment [24]. The results of the present study contradict a study conducted by Cobankara FK et al., which examined the apical seal of four sealers using an automated fluid filtering approach and concluded that Sealapex exhibited minimal leakage compared to AH Plus and ZOE sealers [25].It has been observed from the present study that Epoxy Seal and BioRoot RCS have the maximum amount of marginal adaptation to root dentin, followed by MTA-Fillapex and Sealapex.

This study is subject to specific limitations. During the longitudinal cutting of tooth samples, the gutta-percha may have been inadvertently withdrawn from the canal wall, potentially leading to variations in results. Further, the bond strength was assessed after 10 days of obturation to make sure the sealers were well set and bonded to root dentin. These parameters may not be applicable in vivo conditions as bond strength could potentially reduce over time, thereby increasing the risk of failure. Further in vivo studies may be conclusive in corroborating the results of this study.

## Conclusions

Within the limitations of this study, it is concluded that both EpoxySeal and BioRoot RCS exhibited superior marginal adaptation to root dentin. Marginal adaptation plays a pivotal role in the success of endodontic treatments by facilitating effective filling of the interface between root canal walls and filling material, thus minimizing the risk of bacterial infiltration and subsequent infection. However, BioRoot RCS is recommended as the superior sealer due to its additional beneficial properties. Notably, its antimicrobial activity in eradicating residual bacteria within the root canal system reduces the potential for post-treatment infections and enhances the overall success rate of endodontic procedures. Another significant advantage of BioRoot RCS lies in its hydrophilic affinity during setting. This trait enables the sealer to interact more effectively with the natural moisture present in the root canal, facilitating better penetration into the dentinal tubules and micro-irregularities within the canal walls. Consequently, BioRoot RCS achieves a tighter and more reliable seal, which is essential for preventing microleakage and ensuring the durability of the root canal filling. These attributes collectively contribute to its superior performance in maintaining a sterile environment and achieving a secure seal, thereby promoting the long-term success of endodontic treatments.
